# Another Example of Conditioned Taste Aversion: Case of Snails

**DOI:** 10.3390/biology9120422

**Published:** 2020-11-26

**Authors:** Junko Nakai, Yuki Totani, Dai Hatakeyama, Varvara E. Dyakonova, Etsuro Ito

**Affiliations:** 1 Department of Biology, Waseda University, Tokyo 162-8480, Japan; kbc-jun5211@ruri.waseda.jp (J.N.); y.totani@fuji.waseda.jp (Y.T.); 2 Faculty of Pharmaceutical Sciences, Tokushima Bunri University, Tokushima 770-8514, Japan; daihatake926@ph.bunri-u.ac.jp; 3 Koltzov Institute of Developmental Biology, Russian Academy of Sciences, Moscow 119334, Russia; dyakonova.varvara@gmail.com; 4 Graduate Institute of Medicine, School of Medicine, Kaohsiung Medical University, Kaohsiung 80756, Taiwan

**Keywords:** conditioned taste aversion, insulin, long-delay learning, *Lymnaea stagnalis*, selective associability, single-trial learning

## Abstract

**Simple Summary:**

It is important to decide what to eat and what not to eat in the life. Children are likely to reject new foods. When eating a new food results in a negative experience, the child will avoid that specific food in the future. This phenomenon is called ‘conditioned taste aversion’ in mammals, and it is considered necessary for survival by preventing subsequent ingestion of sickening foods. Many researchers study the same kind of phenomenon in invertebrates, too. For example, the formation of conditioned taste aversion was found in the pond snail, *Lymnaea stagnalis,* with the selective associability between a sweet sucrose solution and a bitter KCl solution. A sweet food attracts many kinds of animals, resulting in the feeding response, whereas a KCl solution is an aversive stimulus, inducing a withdrawal response in snails. After repeated temporally-contingent presentations of these two stimuli, the sucrose solution no longer elicits a feeding response, and this phenomenon persists for a long term. In the present review, we first outline the mechanisms of conditioned taste aversion in mammals, then introduce the conditioned taste aversion in snails, and compare them. Furthermore, the molecular events in snails are discussed, suggesting the general mechanism in conditioned taste aversion.

**Abstract:**

Conditioned taste aversion (CTA) in mammals has several specific characteristics: (1) emergence of a negative symptom in subjects due to selective association with a taste-related stimulus, (2) robust long-term memory that is resistant to extinction induced by repeated presentation of the conditioned stimulus (CS), (3) a very-long-delay presentation of the unconditioned stimulus (US), and (4) single-trial learning. The pond snail, *Lymnaea stagnalis,* can also form a CTA. Although the negative symptoms, like nausea, in humans cannot be easily observed in invertebrate animal models of CTA, all the other characteristics of CTA seem to be present in snails. Selective associability was confirmed using a sweet sucrose solution and a bitter KCl solution. Once snails form a CTA, repeated presentation of the CS does not extinguish the CTA. A long interstimulus interval between the CS and US, like in trace conditioning, still results in the formation of a CTA in snails. Lastly, even single-trial learning has been demonstrated with a certain probability. In the present review, we compare, in detail, CTA in mammals and snails, and discuss the possible molecular events in CTA.

## 1. Introduction

Humans tend to reject new and unfamiliar food items. For example, before eating the local cuisine when visiting a foreign country, we may first confirm whether the food item is hot, bitter, or sweet by querying the local residents. Children are especially likely to reject new foods. When eating a new food results in a negative experience, such as an upset stomach or diarrhea, the child will strongly avoid that specific food in the future. It does not matter if the food is safe to eat or poisonous—many people have eaten too many foods that resulted an upset stomach during childhood; the offending foods are subsequently avoided, even in adulthood. This phenomenon is called ‘conditioned taste aversion (CTA)’ [1,2,3], because the taste of the food becomes aversive. CTA has been vigorously studied in rats (Section 2).

Findings regarding various types of CTA have revealed the following common characteristics: (1) CTA is considered necessary for survival by preventing subsequent ingestion of sickening foods. This ability is considered an adaptive trait or survival mechanism by which the body avoids ingesting sickening substances before they can cause harm, resulting in robust maintenance of long-term memory for the CTA. (2) CTA can be formed by an associative learning protocol. In this case, a conditioned stimulus (CS) is a novel taste of a new foodstuff, whereas an unconditioned stimulus (US) is a sickening food, causing, for example, nausea or diarrhea. We here consider the definition of the words, CS and US [4]. In Pavlovian conditionings, the animal is presented with a stimulus that evokes a certain behavioral response. This stimulus, which is chosen and controlled by the experimenter, is named US. The response to the US is the unconditioned response (UR). Another stimulus, neutral or almost neutral with respect to the UR, is then presented in association with the US. This stimulus is called CS. The CS is also chosen and controlled by the experimenter. The association between the CS and US alters the response to the CS, resulting in a conditioned response (CR), which is similar to the UR. According to this definition, the use of CS and US seems odd in CTA, because the food CS is not neutral for the UR or CR that demonstrates that subjects do not eat the food. However, according to the precedents of many textbooks and articles written for CTA [5] and in order to avoid confusion in future CTA studies, we use the CS and US for a novel taste of food and a sickening food, respectively, in the present review. (3) CTA has selectivity for novel tastes. If a subject has experienced a taste previously, it can no longer successfully serve as a key taste for CTA. This is called ‘selective associability and latent inhibition’ (see below). Subjects may fail to consciously recognize a connection between the perceived taste (CS, i.e., the cause) and the sickness (conditioned response, i.e., the effect). (4) The interval between the CS-US pairing can be very long, and still results in CTA. That is, CTA is thought to be a type of a trace conditioning, called ‘long-delay learning’. (5) CTA may be formed by ‘single-trial learning’. For an animal’s survival, it is reasonable that animals can form a CTA by single-trial learning or at least several trials of learning, because single-trial learning reduces the probability of consuming the same substance in the future, avoiding further poisoning.

In the present review, we first outline the mechanisms of CTA in mammals, then introduce CTA in snails, and compare them. Furthermore, we discuss the molecular events in snails, and suggest a general mechanism for CTA. Our aim with this review is to convince the reader that invertebrate studies are very useful for studying the mechanisms underlying mammalian learning and memory.

## 2. Conditioned Taste Aversion in Mammals

**Figure 1:**
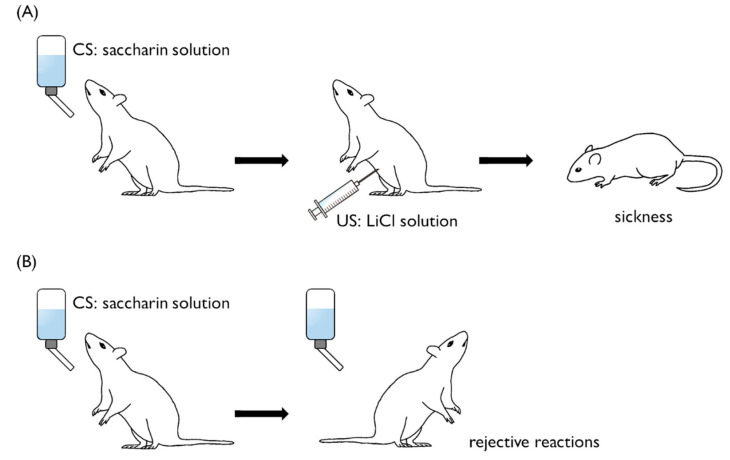
Training protocol of conditioned taste aversion in mammals. (**A**) Rats receive a saccharin solution (CS) followed by injection of LiCl (US). NaCl is used as a control solution for LiCl. (**B**) After CS–US pairing, rats injected with LiCl following access to a saccharin solution consume significantly less saccharin solution than NaCl-injected controls.

As explained in the Introduction, CTA in animals is a type of associative learning [6,7]. The common experimental approach to studying mammalian CTA is intraperitoneal injection of a LiCl solution (i.e., US) after consumption of a palatable, novel taste like a sweet saccharin solution (i.e., CS) (Figure 1) [8]. LiCl elicits hypothermia and a decreased heart rate in rats. After a novel, sweet saccharin taste has been paired with LiCl, re-exposure to the sweet saccharin taste alone induces these same negative responses [9,10]. In the 1950s, John Garcia demonstrated CTA for saccharin resulting from exposure to gamma radiation in rats [11,12]. This effect is thus called the ‘Garcia effect’. In humans, the negative sensation is observed as nausea.

Here, we review the following three major characteristics of mammalian CTA—selective associability and latent inhibition, long-delay learning, and single-trial learning.

### 2.1. Selective Associability and Latent Inhibition

The first characteristic of mammalian CTA is that it depends on the pairing between a specific cue (i.e., taste) and a specific event (i.e., nausea) [13]. This is called ‘selective associability’ [14]. In Pavlovian conditioning, all neutral stimuli (e.g., taste, sound, or light) are thought to be equivalent for use as the CS, and thus, due to this characteristic, CTA defies Pavlovian conditioning. In addition, CTA is reported to continue for tens of years [15], and this type of learning is considered to be very robust. In other words, CTA strongly resists extinction. A rebuttal of this view is that subjects who acquire a CTA simply avoid the CS. That is, the CS is not perceived by the subjects during the period in which the CTA exists because they refuse to sample it again. For example, when subjects are forced to drink water containing the CS (e.g., a saccharin solution), extinction of the CTA occurred at almost the same rate as other types of classical conditioning [16,17]. For a CS to be effective in CTA, the subject should not be exposed to the CS prior to training. If a foodstuff has been previously tried and found to be non-aversive, it is thereafter no longer considered a toxic agent. This phenomenon is called ‘latent inhibition’ [18,19].

### 2.2. Long-Delay Learning

Trace conditioning is a type of Pavlovian conditioning in which there is a gap in time between the termination of the CS and the onset of the US. In CTA, trace conditioning forms even if the interval between the CS and US is very long. This is called ‘long-delay learning’ [20]. In Garcia’s study in 1966, a saccharin solution was used as the CS and an injection of apomorphine was used as the US. The long-delay application of apomorphine after the CS was confirmed to form a CTA for up to 75 min. Further, Etscorn and Stephens showed that when an anticancer agent, cyclophosphamide, was used as the US, a CTA formed even with a 24-h delay before administering the US. Both apomorphine and cyclophosphamide induced nausea [21]. Moreover, another observation was reported for the interval between the CS and US. Schafe and colleagues concluded that close temporal contiguity between the CS and US is not required for CTA acquisition [22], even though general Pavlovian conditioning needs it.

### 2.3. Single-Trial Learning

In the experiments by Etscorn and Stephens, the 24-h delayed learning was formed following a single pairing of the CS and US (i.e., single-trial learning) [21]. Single-trial learning was also observed in many other studies [23]. Single-trial learning is useful for studying the brain regions involved in CTA. Because the training protocol comprises a single trial, the expression pattern of immediate early genes (i.e., genes that are activated transiently and rapidly in response to a variety of stimuli) can be clearly observed. In rats, the expression of immediate early genes, such as *c-fos*, in the brain can be analyzed to reveal the regions active during the formation of a CTA [24]. Furthermore, the studies of second-order conditioning and sensory preconditioning were achieved in mammalian CTA [25]. These complicated conditionings were confirmed by the use of various stimuli [26].

## 3. Molecular Events in Mammalian CTA

Molecular events in mammalian CTA remain poorly understood, even though a long period has been spent studying them [27]. Barki-Harrington et al. [28] well summarized the profiles of the following three molecular aspects: (1) the role of neurotransmitters, glutamate and acetylcholine, and their receptors; (2) the role of translation regulation in memory consolidation; and (3) the role of expression of immediate early genes. Almost all studies were performed based on those of long-term potentiation and other ordinary studies of learning and memory. For (1), neurotransmitters, a significant release in glutamate was observed following LiCl injection [29]. When glutamate was injected into the basolateral amygdala (BLA), CTA was enhanced [30]. Blockade of glutamate receptors between the ingestion of the saccharin (i.e., CS) and administration of the malaise-inducing LiCl (i.e., US) impaired CTA acquisition [31]. Formation of CTA memory was eliminated when a muscarinic receptor antagonist (scopolamine or atropine) was injected into the insular cortex after CTA acquisition [32]. As expected in (2), translation for de novo protein synthesis required for memory consolidation, an injection of a protein synthesis inhibitor (cycloheximide or anisomycin) impaired CTA [33]. For (3), the expression of immediate early genes, the monitor of c-Fos mRNA and protein levels was performed to identify the active brain regions for CTA. Many research groups found that c-Fos expression was observed in the BLA and the insular cortex after a few hours of CTA formation [34,35]. The synaptic underpinnings in the BLA and the insular cortex have been recently clarified [36].

As discussed in Section 7, we consider the downstream cascades of insulin reception. Some important molecules are targeted there, and one of them is phosphoinositide-3-kinase (PI3K). In mammalian CTA, microinfusion of brain-derived neurotrophic factor (BDNF) induced a lasting potentiation of synaptic efficacy in the insular cortex, modifying the CTA retention [37], and this effect was dependent on both the activation of mitogen-activated protein kinase (MAPK) and PI3K in the insular cortex [38]. At present, the function of PI3K may be intermediated between mammalian CTA and snail CTA.

Very recently, the role of EphB2 (Eph family type-B receptor 2) on mammalian CTA memory formation was studied [39]. EphB2 is known to be required for normal brain development and function [40]. Using wild-type mice and age-matched EphB2^−/−^ and EphB2^lacZ/lacZ^ mice, Alapin et al. [39] showed that long-term CTA memory was intact in young mice that lacked EphB2 (i.e., EphB2^−/−^) or in young mice with no EphB2 forward signaling (EphB2^lacZ/lacZ^). On the other hand, although long-term CTA memory was enhanced in old mice [41], the ability to form long-term CTA memory was impaired in old EphB2^−/−^ and EphB2^lacZ/lacZ^ mice. Therefore, EphB2 forward signaling is needed for CTA memory formation in old mice. Furthermore, Levitan and colleagues noticed the BLA projection neurons, because the BLA plays a crucial role in mammalian CTA memory as described above [27,30]. Their results suggested that (a) CTA training (i.e., the pairing of saccharin and LiCl) increases the activity of STK11 (serine/threonine kinase 11) and c-Fos (an activity-dependent transcription factor) in BLA projection neurons; (b) CTA increases STK11 protein, resulting in downstream phosphorylation and activation of AMP-related kinases and c-Fos induces the transcription of Fos effector genes; (c) both AMP-related kinases and Fos effectors regulate ion channel activity, reducing intrinsic excitability of BLA projection neurons; and (d) reduced intrinsic excitability in BLA projection neurons plays a key role in CTA memory formation, reducing saccharin consumption in test trials. It is excellent that mammalian CTA studies can be performed using knock-out mice.

## 4. Conditioned Taste Aversion in Snails

**Figure 2:**
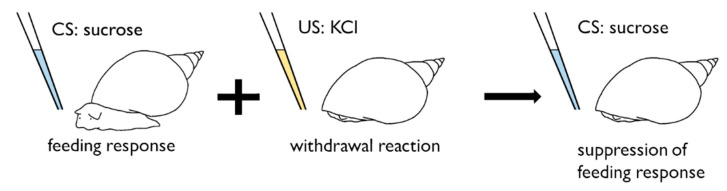
Training protocol of conditioned taste aversion in snails. After a sucrose solution (CS) is paired with a KCl solution (US), a sucrose solution does not elicit a feeding response in trained snails.

CTA also occurs in snails, as first reported by Kojima et al. [42] in 1996, who applied various stimuli to the pond snail, *Lymnaea stagnalis* to induce the formation of aversive and appetitive training and find a ‘selective associability’ between a sweet sucrose solution and a bitter KCl solution. The sucrose solution was the CS and had not been applied previously to snails before training to avoid inducing latent inhibition. Generally, a sweet food is attractive to many animals, resulting in a feeding response (i.e., an increase in the number of bites in response to sucrose). On the other hand, a KCl solution is an aversive stimulus to snails that induces a whole-body withdrawal response. Humans perceive a KCl solution as tasting bitter. Thus, a KCl solution was used as the US [43,44]. In some studies, an electric shock is used as the US instead of KCl [45,46]. In the snail CTA paradigm, the sweet CS was paired with the aversive US (Figure 2). After repeated temporally-contingent presentations of the CS and US, the CS no longer elicited a feeding response, and this CTA persisted for at least a month. The effects of the concentrations of sucrose and KCl and the trial number on the strength of snail CTA have been examined [42,45,47]. The suitable concentrations of these solutions were found [42,45], and a small number of trials (e.g., 1–10 trials) were enough for snail CTA [42,47]. ‘Single-trial learning’ in snails will be described later. The most important issue in this snail CTA is that the negative symptoms, such as nausea in humans and rats as well as nausea in response to meat with a poison, including LiCl, in wild animals like a coyote and a wolf [48], cannot be easily observed. Researchers are now attempting to overcome this issue in various invertebrate species [47,49].

In snail CTA, the key neurons in the feeding circuitry are known–cerebral giant cells (CGCs) and follower neurons belonging to the feeding central pattern generator [50,51,52]. Kojima and colleagues examined the synaptic connection between the CGC, a regulatory neuron of the feeding central pattern generator, and neuron 1 medial (N1M), an interneuron belonging to the central pattern generator [50]. Their results showed that an inhibitory postsynaptic potential (IPSP), which is activated in the CGC and recorded in the N1M, is larger and lasts longer in CTA-trained snails than in control snails, suggesting that this enhanced IPSP in the N1M underlies the suppression of the feeding response in snail CTA [50]. In addition to Kojima’s observation, Otsuka et al. [51] further clarified the mechanism in the feeding central pattern generator. The N1M is thought to be ultimately activated by the CGC via neuron 3 tonic (N3t). That is, the inhibitory monosynaptic inputs from N3t to N1M are facilitated. N1M and N3t are members of the feeding central pattern generator. To examine the involvement of a second messenger, cAMP, Otsuka et al. [51] injected cAMP into the CGC and monitored the potentials of the B3 motor neuron activated by the CGC. The B3 activity was used as an index for synaptic inputs from N3t to N1M. The B3 potentials were transiently enlarged. Thus, when the cAMP concentration is increased in the CGC by CTA training, cAMP-induced changes may play a key role in the establishment of a memory trace in N3t.

In 2017, Sunada and colleagues performed ablation experiments of the CGC somata to further confirm the requirement of the CGC in snail CTA [52]. Their results demonstrated that: (1) the CGC somata are not necessary for learning acquisition, (2) the CGC somata are necessary for memory formation, and (3) the CGC somata are necessary for memory recall. Therefore, these findings confirmed that CGCs function in the long-term memory of CTA in snails. This relatively-simple pathway facilitates the analysis of the cellular mechanisms of CTA in invertebrates.

‘Long-delay learning’ and ‘single-trial learning’ were examined in snails by Nakai et al. [47] and Sugai et al. [53], respectively. When the interstimulus interval between the presentation of the CS and US was examined, the interstimulus interval could be set as long as minutes [47]. No inverted U-shaped relationship, like the relationship of Yerkes–Dodson’s law [54], was observed between the interstimulus interval and the conditioned response (i.e., suppression of feeding). Thus, snail CTA formed even when presentation of the US was delayed. In addition, Nakai et al. [47] examined the time course of the protein synthesis-dependent period during the consolidation of snail CTA to long-term memory by pharmacologic inhibition of transcription or translation. Their results indicated that the protein synthesis-dependent period coincided with the CTA training. That is, de novo protein synthesis to form long-term memory occurs during the same time-frame as long-delay learning for CTA.

In Kojima’s paradigm, snail CTA is formed by presenting some pairings of sucrose as the CS and KCl as the US [42]. As long as the term ‘CTA’ is used for long-term aversive snail learning, then single-trial learning should be examined [53]. In snails, particular measures are used to distinguish between ‘good’ and ‘poor’ performers, similar to a minimum passing mark for students. If a snail possesses long-term memory (i.e., a good performer), it is expected to not open its mouth following the presentation of the CS. Some snails, however, open their mouths by chance in the absence of any delivered stimulus [42]. Such spontaneous mouth openings occur at a rate of about one per minute. Good performers are thus defined as snails that made 0 to 1 bite/min in response to the CS during the post-test sessions, and poor performers are defined as snails that made ≥2 bites/min in response to the CS during the post-test sessions that are performed by a single CS application over time after CTA training. Sugai et al. [53] obtained a constant ratio of poor to good performers for snail CTA, and approximately 40% of CTA-trained snails were good performers following a single-trial learning procedure. The success ratio for single-trial learning is not 100% in snails, but it is important to note that an extremely-small number of US–CS pairings leads to the learning acquisition and memory formation in snails.

## 5. ‘Necessity Knows No Law’ Manner of Snail CTA

**Figure 3:**
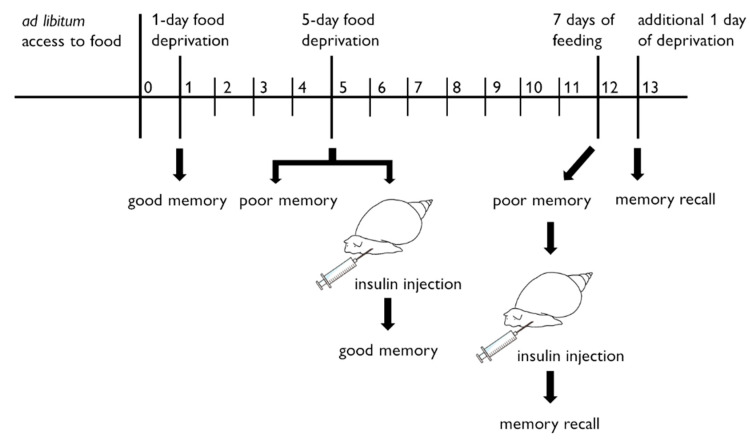
Relation between food deprivation and memory recall in snail conditioned taste aversion (CTA). Snails with ad libitum access to food exhibit poor memory formation. CTA memory is enhanced by 1-day food deprivation, whereas CTA memory is attenuated by 5-day food deprivation. If insulin is injected into the snails after 5 days of food deprivation before the CTA training, the CTA memory is observed. If, after the snails are food deprived for 5 days, they are fed again to keep them healthy for 7 days, they exhibit poor memory. If the snails are again food-deprived for 1 day before memory testing, however, their insulin levels are increased and they recall the CTA memory. On the other hand, if the snails are injected with insulin instead of being subjected to 1-day food deprivation before memory testing, they are able to recall the CTA memory. We have confirmed that an insulin injection decreases hemolymph glucose levels, but the insulin levels and/or the insulin sensitivity are thought to be regulated not only by food intake but also by other factors (Section 6 and Section 7).

In snails, food deprivation for 1 day before CTA training results in optimal learning and consolidation into long-term memory. In snails with 5-day food deprivation before CTA training, however, learning and memory do not form. Ito et al. [55] hypothesized that snails do in fact learn CTA and form a long-term memory of CTA when trained after prolonged 5-day food deprivation, but that such severe food deprivation blocks their ability to express the memory. Their results showed that long-term memory expression occurs in 5-day food-deprived snails that are then fed for 7 days followed by 1 day of food deprivation (Figure 3). Moreover, this CTA and its long-term memory are context-dependent and are observed only when the snails are placed in a context similar to that in which the training occurred. That is, snails are restricted with respect to the ‘necessity knows no law’ concept [56]. In mammalian CTA, as far as we know, such a phenomenon was not observed. However, after rats form CTA, if they are hungry, they intake the CS (i.e., demonstration of extinction) [57]. There is no help for eating in animals.

## 6. Role of Insulin-Related Peptide in Learning and Memory Formation for Snail CTA

In 2006, Azami et al. [58] produced a cDNA microarray for *Lymnaea*, which they used to examine the expression levels of mRNAs in the snail central nervous system. The aim of their study was to investigate the altered gene activity associated with snail CTA. They found that some molluscan insulin-related peptides (MIPs) were upregulated at the mRNA level during CTA. They further analyzed the details of the mRNA level of MIP II (a major MIP) using real-time PCR, and found that the MIP II levels in the central nervous system of snails exhibiting ‘good’ memory for CTA were significantly higher than those in snails exhibiting ‘poor’ memory. Following these results, Murakami and colleagues performed experiments to apply MIPs or mammalian insulin exogenously to an isolated snail central nervous system [59,60,61]. They observed a long-term change in synaptic efficacy (i.e., enhancement) of the synaptic connection between the CGCs and the B1 motor neurons. These findings suggest that MIPs trigger changes in synaptic connectivity that are correlated with the consolidation of CTA into long-term memory in the snail central nervous system.

Mita et al. [62,63] further examined the effects of insulin on CTA memory in snails. As described above, 1-day food deprivation resulted in the best learning and memory for CTA in snails, whereas a more prolonged 5-day food deprivation made the snails incapable of learning or remembering. Their results demonstrated that injecting the snails with insulin to reduce the hemolymph glucose concentration resulted in better learning and memory in the 5-day food-deprived snails, but injecting glucose into 5-day food-deprived snails did not alter their inability to learn and remember. On the basis of these results, we proposed the ‘insulin spike hypothesis’ (i.e., a rise in the insulin concentration in the central nervous system of snails) for the formation of CTA and its long-term memory [64,65,66]. In 2020, Totani et al. [67] provided evidence to support this hypothesis.

In Totani’s experiments, 1-day food-deprived snails showed the best CTA learning and memory, whereas more severely food-deprived snails (i.e., 5-day food-deprivation) did not express good memory [67]. Previous studies by Ito et al. [55], however, showed that CTA and its long-term memory are indeed formed in 5-day food-deprived snails, but memory recall for the CTA is prevented by the effects of food deprivation. Long-term memory recall in 5-day food-deprived snails is expressed following 7 days of feeding and then 1 day of food deprivation. Totani et al. [67] revealed that this 1 day of food deprivation before the memory test in snails increases the MIP II mRNA levels in the central nervous system. Instead of this 1-day food deprivation, injecting insulin into the snails activates CTA neurons and mimics the food deprivation state before the memory test. Together, the results suggest that a spike in insulin release recapitulates the optimal internal state for long-term memory recall following CTA training in snails [67].

## 7. Perspectives in Molecular Events after Insulin Reception

At this stage, we must consider why the insulin levels in the snail central nervous system are altered by 1-day food deprivation. Although we demonstrated this change at the mRNA level [67], at present we do not have any tool to measure the MIP proteins directly. Accordingly, we cannot affirm that only an increase in insulin level affects snail CTA. For example, another possibility is that insulin sensitivity is circadian-rhythm dependent [68,69,70]. Circadian rhythmicity has been confirmed in snails, and affects CTA learning and memory [71]. We should thus consider that both insulin levels and insulin sensitivity may change regardless of food intake in snail CTA.

Studies of insulin function in invertebrates, such as *Caenorhabditis elegans* and *Drosophila*, have also been reported [72,73]. Especially in *Drosophila*, the relation between the actions of insulin and those of cAMP-regulated transcriptional coactivator (CRTC) have been clarified [74]. CRTC is thought to be activated when the insulin-signaling cascade is downregulated in hungry flies [75]. Hungry flies learn conditioned food aversions in one trial because of downregulation of the insulin-signaling pathway and upregulation of the CRTC-signaling pathway [74]. A state of hunger in flies is induced by 9 to 16 h of food deprivation before conditioned food aversion, and this may correspond to the state of mild food deprivation in the studies using snails [76]. The relationship between the insulin signals and the CRTC signals in snails has not yet been studied, and thus the molecular mechanisms should be carefully examined in snails.

**Figure 4:**
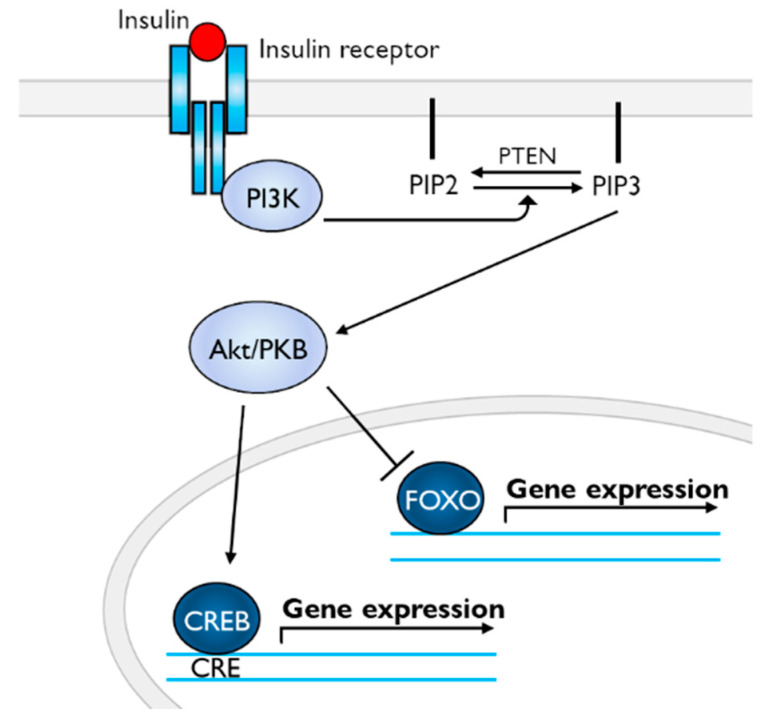
Scheme of deduced insulin-signaling cascades for memory enhancement. Akt/PKB phosphorylates CREB (i.e., CREB1 in *Lymnaea*), resulting in gene expression, whereas Akt/PKB phosphorylates FOXO, inducing its secession from DNA. Abbreviations: PI3K, phosphoinositide-3-kinase; PIP2, phosphatidylinositol 4,5-bisphosphate; PIP3, phosphatidylinositol 3,4,5-trisphosphate; PTEN, phosphatase and tensin homolog deleted on chromosome 10 (i.e., PIP3 phosphatase); Akt/PKB, Akt/protein kinase B: CREB, cAMP response element-binding protein; FOXO, forkhead box O.

Special attention should be focused on the following two kinds of transcription factors in the insulin-signaling cascade for CTA—cAMP response element-binding protein (CREB) and forkhead box O (FOXO) [77] (Figure 4). Insulin binding to its tyrosine kinase receptors drives the phosphoinositide-3-kinase-Akt/protein kinase B (PI3K-Akt/PKB) pathway [78]. The PI3K-Akt/PKB pathway regulates CREB for gene expression [79,80,81]. In snails, activation of CREB plays a necessary role in the formation of long-term memory [82,83,84,85,86]. At the mRNA level, the amount of suppressor CREB (i.e., CREB2) is more abundant than the amount of activator CREB (i.e., CREB1) [87]. The other transcription factor, FOXO, is also activated by the PI3K-Akt/PKB pathway [80,88,89]. Interestingly, FOXO is involved in learning and memory formation in a starved state [90].

## 8. Role of Insulin in Mammalian Brain

Finally, the importance of insulin-signaling cascades in memory formation in the mammalian brain is also discussed. Almost all the articles about the function of insulin for memory in mammals are involved in the studies of the relationship between type 2 diabetes and Alzheimer’s disease [91,92]. The common feature of these two diseases is insulin resistance, and thus some antidiabetic drugs, such as intranasal insulin, pioglitazone, metformin, and liraglutide, are promising drugs that could be useful in the treatment of Alzheimer’s disease [93]. The roles of insulin in the mammalian brain are considered as follows [94]: (1) proteostasis influencing clearance of the amyloid β peptide and phosphorylation of tau, which are hallmarks of Alzheimer’s disease, and (2) modulation of vascular function through the effects on vasoreactivity, lipid metabolism, and inflammation. Another idea showed that these two diseases (i.e., type 2 diabetes and Alzheimer’s disease) may be linked with heat-shock proteins [95]. Anyhow, the detailed molecular events of insulin cascades in the mammalian brain should be examined in the future.

In addition, a small number of studies have been reported for a role of insulin in depression [96]. Insulin has an influence on hormones involved in the hypothalamic–pituitary–adrenal axis (HPA) negative feedback system [97]. Major depression induces an increase in the secretion of corticotropin-releasing hormone, the overproduction of glucocorticoids, the impairment of the sensitivity of glucocorticoid receptor, and the damage of HPA negative feedback mechanism [98]. HPA axis dysfunction is associated with insulin action in patients with depression [99]. As shown here, the role of insulin in the mammalian brain is mainly examined in Alzheimer’s disease and depression, but not in CTA.

## 9. Conclusions

In the present review, we compared the characteristics of CTA between mammals and snails. Studies of behavioral, cellular, and molecular events in snails can be very informative for studies of these events in mammalian CTA [100]. Only a few points in snail CTA do not meet the conditions of mammalian CTA. Furthermore, it is important to note that snail CTA depends on the state of food deprivation. Food deprivation for 1 day in snails before CTA training results in the best learning and memory formation, whereas 5-day food deprivation before CTA training prevents learning and memory formation [55]. In mammals, short-term fasting (i.e., comparable to 1-day food deprivation in snails) is thought to enhance cognition, including memory consolidation [101,102]. In contrast, obesity impairs cognition and increases the risk of dementia in humans [103,104]. Snail CTA studies may also provide some insight into the advantages of short-term fasting.

## References

[1] Schafe G.E., Bernstein I.L., Capaldi E.D. (1996). Taste aversion learning. Why We Eat What We Eat: The Psychology of Eating.

[2] Reilly S., Schachtman T.R. (2008). Conditioned Taste Aversion: Behavioral and Neural Processes.

[3] Chambers K.C. (2018). Conditioned taste aversions. World J. Otorhinolaryngol. Head Neck Surg..

[4] Dudai Y. (1989). The Neurobiology of Memory.

[5] Freeman K.B., Riley A.L., Reilly S., Schachtman T.R. (2009). The Origins of Conditioned Taste Aversion Learning: A Historical Analysis. Conditioned Taste Aversion, Behavioral and Neural Processes.

[6] Bures J., Bermudez-Rattoni F., Yamamoto T. (1998). Conditioned Taste Aversion: Memory of a Special Kind.

[7] Nakajima S. (2020). Effect of pretrial running on running-based taste aversion learning in rats. J. Exp. Psychol. Anim. Learn. Cogn..

[8] Karniol I.G., Dalton J., Lader M.H. (1978). Acute and chronic effects of lithium chloride on physiological and psychological measures in normals. Psychopharmacology.

[9] Meachum C.L., Bernstein I.L. (1990). Conditioned responses to a taste conditioned stimulus paired with lithium chloride administration. Behav. Neurosci..

[10] Bull D., Brown R., King M., Husband A. (1991). Modulation of body temperature through taste aversion conditioning. Physiol. Behav..

[11] Garcia J., Kimeldorf D.J., Koelling R.A. (1955). Conditioned aversion to saccharin resulting from exposure to gamma radiation. Science.

[12] Garcia J., Lasiter P.S., Bermudez-Rattoni F., Deems D.A. (1985). A General Theory of Aversion Learning. Ann. N. Y. Acad. Sci..

[13] Garcia J., Koelling R.A. (1966). Relation of cue to consequence in avoidance learning. Psychon. Sci..

[14] Bevins R.A. (1992). Selective Associations: A Methodological Critique. Psychol. Rec..

[15] Garb J.L., Stunkard A.J. (1974). Taste aversions in man. Am. J. Psychiatry.

[16] Spector A.C., Smith J.C., Hollander G.R. (1981). A comparison of dependent measures used to quantify radiation-induced taste aversion. Physiol. Behav..

[17] Spector A.C., Smith J.C., Hollander G.R. (1983). The effect of postconditioning CS experience on recovery from radiation-induced taste aversion. Physiol. Behav..

[18] Revusky S.H., Bedarf E.W. (1967). Association of Illness with Prior Ingestion of Novel Foods. Science.

[19] Kalat J.W., Rozin P. (1973). “Learned safety” as a mechanism in long-delay taste-aversion learning in rats. J. Comp. Physiol. Psychol..

[20] Garcia J., Ervin F.R., Koelling R.A. (1966). Learning with prolonged delay of reinforcement. Psychon. Sci..

[21] Etscorn F., Stephens R. (1973). Establishment of conditioned taste aversions with a 24-h CS-US interval. Physiol. Psychol..

[22] Schafe G.E., Sollars S.I., Bernstein I.L. (1995). The CS–US interval and taste aversion learning: A brief look. Behav. Neurosci..

[23] Garcia J., Hankins W.G., Rusiniak K.W. (1974). Behavioral Regulation of the Milieu Interne in Man and Rat. Science.

[24] Swank M.W., Bernstein I.L. (1994). c-Fos induction in response to a conditioned stimulus after single trial taste aversion learning. Brain Res..

[25] Schachtman T.R., Ramsey A., Pineño O., Reilly S., Schachtman T.R. (2009). Postconditioning event manipulations on processing of the target conditioned stimulus in conditioned taste aversion. Conditioned Taste Aversion, Behavioral and Neural Processes.

[26] Dwyer D.M., Burgess K.V., Honey R.C. (2012). Avoidance but not aversion following sensory preconditioning with flavors: A challenge to stimulus substitution. J. Exp. Psychol. Anim. Behav. Process.

[27] Levitan D., Liu C., Yang T., Shima Y., Lin J.-Y., Wachutka J., Marrero Y., Ali Marandi Ghoddousi R., da Veiga Beltrame E., Richter T.A. (2020). Deletion of Stk11 and Fos in mouse BLA projection neurons alters intrinsic excitability and impairs formation of long-term aversive memory. eLife.

[28] Barki-Harrington L., Belelovsky K., Doron G., Rosenblum K., Reilly S., Schachtman T.R. (2009). Molecular mechanisms of taste learning in the insular cortex and amygdala. Conditioned Taste Aversion, Behavioral and Neural Processes.

[29] Miranda M.-I., Ferreira G., Ramírez-Lugo L., Bermudez-Rattoni F. (2002). Glutamatergic activity in the amygdala signals visceral input during taste memory formation. Proc. Natl. Acad. Sci. USA.

[30] Ferreira G., Miranda M.-I., De La Cruz V., Rodríguez-Ortiz C., Bermúdez-Rattoni F. (2005). Basolateral amygdala glutamatergic activation enhances taste aversion through NMDA receptor activation in the insular cortex. Eur. J. Neurosci..

[31] Jiménez B., Tapia R. (2004). Biochemical modulation of NMDA receptors: Role in conditioned taste aversion. Neurochem. Res..

[32] Parkes S.L., De La Cruz V., Bermudez-Rattoni F., Coutureau E., Ferreira G. (2014). Differential role of insular cortex muscarinic and NMDA receptors in one-trial appetitive taste learning. Neurobiol. Learn. Mem..

[33] Levitan D., Gal-Ben-Ari S., Heise C., Rosenberg T., Elkobi A., Inberg S., Sala C., Rosenblum K. (2016). The differential role of cortical protein synthesis in taste memory formation and persistence. NPJ Sci. Learn..

[34] Lamprecht R., Dudai Y. (1995). Differential modulation of brain immediate early genes by intraperitoneal LiCl. Neuroreport.

[35] Yiannakas A., Rosenblum K. (2017). The Insula and Taste Learning. Front. Mol. Neurosci..

[36] Haley M.S., Bruno S., Fontanini A., Maffei A. (2020). LTD at amygdalocortical synapses as a novel mechanism for hedonic learning. eLife.

[37] Escobar M.L., Figueroa-Guzmán Y., Gómez-Palacio-Schjetnan A. (2003). In vivo insular cortex LTP induced by brain-derived neurotrophic factor. Brain Res..

[38] Castillo D.V., Escobar M.L. (2011). A role for MAPK and PI-3K signaling pathways in brain-derived neurotrophic factor modification of conditioned taste aversion retention. Behav. Brain Res..

[39] Alapin J.M., Dines M., Lamprecht R. (2020). EphB2 receptor forward signaling is needed for normal long-term memory formation in aged mice. Neurobiol. Aging.

[40] Dun X.-P., Parkinson D.B. (2020). Classic axon guidance molecules control correct nerve bridge tissue formation and precise axon regeneration. Neural Regen. Res..

[41] Springer A.D., Fraley S.M. (1981). Extinction of a conditioned taste aversion in young, mid-aged, and aged C57/BL6 mice. Behav. Neural Biol..

[42] Kojima S., Yamanaka M., Fujito Y., Ito E. (1996). Differential Neuroethological Effects of Aversive and Appetitive Reinforcing Stimuli on Associative Learning in *Lymnaea stagnalis*. Zool. Sci..

[43] Ito E., Kobayashi S., Kojima S., Sadamoto H., Hatakeyama D. (1999). Associative Learning in the Pond Snail, *Lymnaea stagnalis*. Zool. Sci..

[44] Ito E., Kojima S., Lukowiak K., Sakakibara M. (2013). From likes to dislikes: Conditioned taste aversion in the great pond snail (*Lymnaea stagnalis*). Can. J. Zool..

[45] Ito E., Yamagishi M., Takigami S., Sakakibara M., Fujito Y., Lukowiak K. (2015). The Yerkes-Dodson law and appropriate stimuli for conditioned taste aversion in *Lymnaea*. J. Exp. Biol..

[46] Totani Y., Kotani S., Odai K., Ito E., Sakakibara M. (2020). Real-Time Analysis of Animal Feeding Behavior With a Low-Calculation-Power CPU. IEEE Trans. Biomed. Eng..

[47] Nakai J., Totani Y., Kojima S., Sakakibara M., Ito E. (2020). Features of behavioral changes underlying conditioned taste aversion in the pond snail *Lymnaea stagnalis*. Invertebr. Neurosci..

[48] Gustavson C.R., Gustavson J.C. (1985). Predation Control Using Conditioned Food Aversion Methodology: Theory, Practice, and Implications. Ann. N. Y. Acad. Sci..

[49] Lai Y., Despouy E., Sandoz J.-C., Su S., de Brito Sanchez M.G., Giurfa M. (2020). Degradation of an appetitive olfactory memory via devaluation of sugar reward is mediated by 5-HT signaling in the honey bee. Neurobiol. Learn. Mem..

[50] Kojima S., Nanakamura H., Nagayama S., Fujito Y., Ito E. (1997). Enhancement of an inhibitory input to the feeding central pattern generator in *Lymnaea stagnalis* during conditioned taste-aversion learning. Neurosci. Lett..

[51] Otsuka E., Matsunaga M., Okada R., Yamagishi M., Okuta A., Lukowiak K., Ito E. (2013). Increase in cyclic AMP concentration in a cerebral giant interneuron mimics part of a memory trace for conditioned taste aversion of the pond snail. Biophysics.

[52] Sunada H., Lukowiak K., Ito E. (2017). Cerebral Giant Cells are Necessary for the Formation and Recall of Memory of Conditioned Taste Aversion in *Lymnaea*. Zool. Sci..

[53] Sugai R., Azami S., Shiga H., Watanabe T., Sadamoto H., Kobayashi S., Hatakeyama D., Fujito Y., Lukowiak K., Ito E. (2007). One-trial conditioned taste aversion in *Lymnaea*: Good and poor performers in long-term memory acquisition. J. Exp. Biol..

[54] Yerkes R.M., Dodson J.D. (1908). The relation of strength of stimulus to rapidity of habit-formation. J. Comp. Neurol. Psychol..

[55] Ito E., Yamagishi M., Hatakeyama D., Watanabe T., Fujito Y., Dyakonova V., Lukowiak K. (2015). Memory block: A consequence of conflict resolution. J. Exp. Biol..

[56] Ito E., Totani Y., Oike A. (2017). Necessity knows no law in a snail. Eur. Zool. J..

[57] Mikulka P., Klein S. (1980). Resistance to Extinction of a Taste Aversion: Effects of Level of Training and Procedures Used in Acquisition and Extinction. Am. J. Psychol..

[58] Azami S., Wagatsuma A., Sadamoto H., Hatakeyama D., Usami T., Fujie M., Koyanagi R., Azumi K., Fujito Y., Lukowiak K. (2006). Altered gene activity correlated with long-term memory formation of conditioned taste aversion in *Lymnaea*. J. Neurosci. Res..

[59] Murakami J., Okada R., Sadamoto H., Kobayashi S., Mita K., Sakamoto Y., Yamagishi M., Hatakeyama D., Otsuka E., Okuta A. (2013). Involvement of Insulin-Like Peptide in Long-Term Synaptic Plasticity and Long-Term Memory of the Pond Snail *Lymnaea stagnalis*. J. Neurosci..

[60] Murakami J., Okada R., Fujito Y., Sakakibara M., Lukowiak K., Ito E. (2013). Paired pulse ratio analysis of insulin-induced synaptic plasticity in the snail brain. J. Exp. Biol..

[61] Hatakeyama D., Okuta A., Otsuka E., Lukowiak K., Ito E. (2013). Consolidation of long-term memory by insulin in *Lymnaea* is not brought about by changing the number of insulin receptors. Commun. Integr. Biol..

[62] Mita K., Okuta A., Okada R., Hatakeyama D., Otsuka E., Yamagishi M., Morikawa M., Naganuma Y., Fujito Y., Dyakonova V. (2014). What are the elements of motivation for acquisition of conditioned taste aversion?. Neurobiol. Learn. Mem..

[63] Mita K., Yamagishi M., Fujito Y., Lukowiak K., Ito E. (2014). An increase in insulin is important for the acquisition conditioned taste aversion in *Lymnaea*. Neurobiol. Learn. Mem..

[64] Kojima S., Sunada H., Mita K., Sakakibara M., Lukowiak K., Ito E. (2015). Function of insulin in snail brain in associative learning. J. Comp. Physiol. A.

[65] Aonuma H., Totani Y., Kaneda M., Nakamura R., Watanabe T., Hatakeyama D., Dyakonova V.E., Lukowiak K., Ito E. (2018). Effects of 5-HT and insulin on learning and memory formation in food-deprived snails. Neurobiol. Learn. Mem..

[66] Totani Y., Aonuma H., Oike A., Watanabe T., Hatakeyama D., Sakakibara M., Lukowiak K., Ito E. (2019). Monoamines, Insulin and the Roles They Play in Associative Learning in Pond Snails. Front. Behav. Neurosci..

[67] Totani Y., Nakai J., Dyakonova V., Lukowiak K., Sakakibara M., Ito E. (2020). Induction of LTM following an Insulin Injection. eNeuro.

[68] Jarrett R.J., Keen H. (1969). Diurnal Variation of Oral Glucose Tolerance: A Possible Pointer to the Evolution of Diabetes Mellitus. BMJ.

[69] Van Cauter E., Polonsky K.S., Scheen A.J. (1997). Roles of Circadian Rhythmicity and Sleep in Human Glucose Regulation. Endocr. Rev..

[70] Mattson M.P. (2019). An Evolutionary Perspective on Why Food Overconsumption Impairs Cognition. Trends Cogn. Sci..

[71] Wagatsuma A., Sugai R., Chono K., Azami S., Hatakeyama D., Sadamoto H., Ito E. (2004). The early snail acquires the learning. Comparison of scores for conditioned taste aversion between morning and afternoon. Acta Biol. Hung..

[72] Kim S.Y., Webb A.E. (2017). Neuronal functions of FOXO/DAF-16. Nutr. Health Aging.

[73] Nässel D.R., Zandawala M. (2019). Recent advances in neuropeptide signaling in Drosophila, from genes to physiology and behavior. Prog. Neurobiol..

[74] Hirano Y., Saitoe M. (2013). Hunger and memory; CRTC coordinates long-term memory with the physiological state, hunger. Commun. Integr. Biol..

[75] Altarejos J.Y., Montminy M. (2011). CREB and the CRTC co-activators: Sensors for hormonal and metabolic signals. Nat. Rev. Mol. Cell Biol..

[76] Totani Y., Nakai J., Ito E. (2020). Impact of insulin on memory recall. J. Data Min. Genom. Proteom..

[77] Totani Y., Nakai J., Hatakeyama D., Ito E. (2020). Memory-enhancing effects of short-term fasting. Eur. Zool. J..

[78] Belfiore A., Frasca F., Pandini G., Sciacca L., Vigneri R. (2009). Insulin Receptor Isoforms and Insulin Receptor/Insulin-Like Growth Factor Receptor Hybrids in Physiology and Disease. Endocr. Rev..

[79] Du K., Montminy M. (1998). CREB Is a Regulatory Target for the Protein Kinase Akt/PKB. J. Biol. Chem..

[80] Kato S., Ding J., Du K. (2007). Differential activation of CREB by Akt1 and Akt2. Biochem. Biophys. Res. Commun..

[81] Fernandez A.M., Torres-Alemán I. (2012). The many faces of insulin-like peptide signalling in the brain. Nat. Rev. Neurosci..

[82] Ribeiro M.J., Serfozo Z., Papp A., Kemenes I., O’Shea M., Yin J.C.P., Benjamin P.R., Kemenes G. (2003). Cyclic AMP response element-binding (CREB)-like proteins in a molluscan brain: Cellular localization and learning-induced phosphorylation. Eur. J. Neurosci..

[83] Sadamoto H., Sato H., Kobayashi S., Murakami J., Aonuma H., Ando H., Fujito Y., Hamano K., Awaji M., Lukowiak K. (2004). CREB in the pond snail *Lymnaea stagnalis*: Cloning, gene expression, and function in identifiable neurons of the central nervous system. J. Neurobiol..

[84] Wagatsuma A., Azami S., Sakura M., Hatakeyama D., Aonuma H., Ito E. (2006). De Novo synthesis of CREB in a presynaptic neuron is required for synaptic enhancement involved in memory consolidation. J. Neurosci. Res..

[85] Sadamoto H., Kitahashi T., Fujito Y., Ito E. (2010). Learning-dependent gene expression of CREB1 isoforms in the molluscan brain. Front. Behav. Neurosci..

[86] Sadamoto H., Saito K., Muto H., Kinjo M., Ito E. (2011). Direct Observation of Dimerization between Different CREB1 Isoforms in a Living Cell. PLoS ONE.

[87] Wagatsuma A., Sadamoto H., Kitahashi T., Lukowiak K., Urano A., Ito E. (2005). Determination of the exact copy numbers of particular mRNAs in a single cell by quantitative real-time RT-PCR. J. Exp. Biol..

[88] Horwood J.M., Dufour F., Laroche S., Davis S. (2006). Signalling mechanisms mediated by the phosphoinositide 3-kinase/Akt cascade in synaptic plasticity and memory in the rat. Eur. J. Neurosci..

[89] Hay N. (2011). Interplay between FOXO, TOR, and Akt. Biochim. Biophys. Acta.

[90] Nagashima T., Iino Y., Tomioka M. (2019). DAF-16/FOXO promotes taste avoidance learning independently of axonal insulin-like signaling. PLoS Genet..

[91] Arvanitakis Z., Tatavarthy M., Bennett D.A. (2020). The Relation of Diabetes to Memory Function. Curr. Neurol. Neurosci. Rep..

[92] De Sousa R.A.L., Harmer A.R., Freitas D.A., Mendonça V.A., Lacerda A.C.R., Leite H.R. (2020). An update on potential links between type 2 diabetes mellitus and Alzheimer’s disease. Mol. Biol. Rep..

[93] Muñoz-Jiménez M., Zaarkti A., García-Arnés J.A., García-Casares N. (2020). Antidiabetic Drugs in Alzheimer’s Disease and Mild Cognitive Impairment: A Systematic Review. Dement. Geriatr. Cogn. Disord..

[94] Kellar D., Craft S. (2020). Brain insulin resistance in Alzheimer’s disease and related disorders: Mechanisms and therapeutic approaches. Lancet Neurol..

[95] Rowles J.E., Keane K.N., Gomes Heck T., Cruzat V., Verdile G., Newsholme P. (2020). Are Heat Shock Proteins an Important Link between Type 2 Diabetes and Alzheimer Disease?. Int. J. Mol. Sci..

[96] Zou X.H., Sun L.H., Yang W., Li B.J., Cui R. (2020). Potential role of insulin on the pathogenesis of depression. Cell Prolif..

[97] Ahmad M.H., Fatima M., Mondal A.C. (2019). Role of Hypothalamic-Pituitary-Adrenal Axis, Hypothalamic-Pituitary-Gonadal Axis and Insulin Signaling in the Pathophysiology of Alzheimer’s Disease. Neuropsychobiology.

[98] Menke A. (2019). Is the HPA Axis as Target for Depression Outdated, or Is There a New Hope?. Front. Psychiatry.

[99] Yokoyama K., Yamada T., Mitani H., Yamada S., Pu S., Yamanashi T., Matsumura H., Nakagome K., Kaneko K. (2015). Relationship between hypothalamic–pituitary–adrenal axis dysregulation and insulin resistance in elderly patients with depression. Psychiatry Res..

[100] Sunada H., Totani Y., Nakamura R., Sakakibara M., Lukowiak K., Ito E. (2017). Two Strains of *Lymnaea stagnalis* and the Progeny from Their Mating Display Differential Memory-Forming Ability on Associative Learning Tasks. Front. Behav. Neurosci..

[101] Marosi K., Moehl K., Navas-Enamorado I., Mitchell S.J., Zhang Y., Lehrmann E., Aon M.A., Cortassa S., Becker K.G., Mattson M.P. (2018). Metabolic and molecular framework for the enhancement of endurance by intermittent food deprivation. FASEB J..

[102] Stockman M.-C., Thomas D.D., Burke J., Apovian C.M. (2018). Intermittent Fasting: Is the Wait Worth the Weight?. Curr. Obes. Rep..

[103] Hao S., Dey A., Yu X., Stranahan A.M. (2016). Dietary obesity reversibly induces synaptic stripping by microglia and impairs hippocampal plasticity. Brain Behav. Immun..

[104] Mattson M.P., Longo V.D., Harvie M. (2017). Impact of intermittent fasting on health and disease processes. Ageing Res. Rev..

